# Investigation on Anti‐Diarrheal and Antipyretic Activities of *citrus maxima* Seeds in Swiss Albino Mice Model

**DOI:** 10.1002/fsn3.4631

**Published:** 2024-12-30

**Authors:** Nazratun Noor Maria, Ayesha Akter Jasmin, Umme Tahmida, Saurav Singha, Tanveer Ahsan

**Affiliations:** ^1^ Department of Pharmacy University of Chittagong Chattogram Bangladesh

**Keywords:** anti‐diarrheal, antipyretic, *Citrus maxima*, methanolic extract, seed, Swiss albino

## Abstract

In the Rutaceae family *Citrus maxima* is the biggest among all fruits, tradtionally used for several purposes due to its diverse ethnomedicinal, phytochemical, and pharmacological activities. Different portions of this plant have been used as sedatives and anti‐inflammatory medications, as well as to treat coughs, fevers, asthma, diarrhea, ulcers, and diabetes. There is a scientific potential for the methanolic seed extract to contain bioactive compounds, similar to those found in other parts of the plant. The extract derived from the seeds has already demonstrated potential biological activity. Moreover, there weren't many in vivo and in vitro research works on these plants' seeds. Therefore, the current research work was undertaken to analyze anti‐diarrheal and antipyretic potentials of the methanolic extract of *Citrus maxima* seeds (MCM). To evaluate MCM's effectiveness in treating diarrhea, castor oil‐induced diarrhea model was utilized as an evaluating method. The antipyretic effect in mice was measured using brewer's yeast induced pyrexia model. The MCM at 200 and 400 mg/kg doses markedly reduced hyperpyrexia, induced by yeast. The effect remained persistent up to 4th hour after administration. In the castor oil induced diarrhea model, the methanolic seed extract showed high level of dose dependent inhibition of diarrhea for 4 h following castor oil administration. Further investigation is necessary to elucidate and clarify the specific causative agents responsible for the dose‐dependent effects observed. The findings of the present study suggest that MCM seed extract may represent a promising alternative for the development of innovative anti‐diarrheal and anti‐pyretic compounds.

## Graphical abstract

A novel preliminary research work to find out the pharmacological potentials of *Citrus maxima* seeds on mice model. This research will add a new way towards the discovery of herbal medicine to aid in human life.

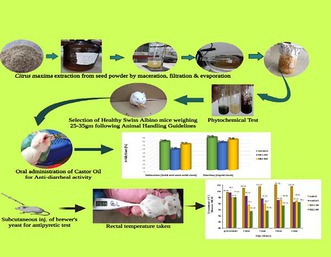

## 1 Introduction

Human life more or less relies on plants. Plant derived foods are incomparable origin of nutrients to human and animals. A number of plant species have enormous medicinal effectiveness for having many bio‐active phytochemical compounds (Joseph and Raj 2010). Research studies have established that taking fruits and vegetables rich diet is linked with a reduced risk of developing different chronic diseases. (Ani and Abel 2018). *Citrus maxima*, belonging to the Rutaceae family, serves as the primary progenitor of the grapefruit (Morton 1987). It is a non‐hybrid natural citrus fruit, habitat to Southeast Asia (Morton 1987). It is commonly known as Pomelo, Pamelo, Pommelo, Jabong, Shaddock etc. and is known as Jambura in Bangladesh. It is commercially cultivated in India, China, Nepal, Thailand, Malaysia, and many other Asian countries. (Lim 2012; Louzada and Ramadugu 2021; Susandarini et al. 2013). The optimal temperature range for the production of citrus fruits falls between 25°C and 30°C, while the coldest month should have a minimum average temperature of at least 15°C. The cessation of growth often occurs at temperatures below 13°C and over 38°C. It is cultivated well in rough sand to heavy clay, but grows well in fertile soils in comparison (Lim 2012; Susandarini et al. 2013). Alot of plant species have significant medicinal activity due to different biologically active phytochemical compounds they possess. Before introducing chemical medicines, man relied on the valuable healing properties of natural medicinal plants. Secondary metabolites, such as saponins, phenols, flavonoids, tannins, terpenoids etc., have been linked to the pharmacological effects of medicinal plants. (Shittu and Abubakar 2014). Several organic compounds are contained by Different portions of this plant. The bark and root of *C. maxima* contain a therapeutically important phytosterol and alkaloid named β‐sitosterol and acridone alkaloid, respectively. *C. maxima* fruits contain an ample amount of polyphenols like naringin, hesperidin, caffeic acid, P‐coumaric acid, ferulic acid, and vanillic acid. The essential oil derived from the unripe fruits and leaves, contains nerolyl acetate, geraniol, nerolol, and limonin (Xu et al. 2008). In folk medicine, the rinds are used for headaches. The leaves of *C. maxima* have medicinally been used as an effective anti‐inflammatory drug (Barrion et al. 2013; KunduSen et al. 2011) by applying a hot leaf decoction to parts of the body that are swollen or inflamed. Studies established that crude extracts of the leaves have hepatoprotective activity, analgesic, anti‐tumor effects, anti‐inflammatory activity, and different effects on central nervous system. The juice of *C. maxima* was found to lower cholesterol and protect the body from damage. Furthermore, it has been discovered that the stem bark portion of the plant exhibits anti‐diabetic properties. Peel was examined for its analgesic effect, antibacterial, hepatoprotective activity, and anti‐inflammatory properties. (Vijaylakshmi and Radha 2015). The aromatic water derived from *Citrus maxima* is utilized for its potential therapeutic effects in alleviating stress and insomnia. (Sohi and Shri 2018). Moreover, the plant is scientifically examined and resulted to possess effective cardiotonic, appetizing, and antitoxic potentials (Arias and Ramón‐Laca 2005).

From a preliminary phytochemical test, it has been found that MCM possess the phytochemicals glycoside, resin, tannin, terpenoids, flavonoids, and alkaloids (Ahsan et al. 2023). Phytochemicals are non‐essential substances that are not necessary for the human body to survive, but they possess significant qualities that can help avoid or combat some prevalent ailments (Holst and Williamson 2008). Moreover, decoctions of the leaves, flowers, fruits, and seeds has folk medicinal characteristics that has been used to cure coughs, fevers, and gastrointestinal ailments in the Philippines and Southeast Asia (Sawant and Panhekar 2017). The seeds are used against coughs, dyspepsia and lumbago while gum exudes remedies for cough in Brazil (Sawant and Panhekar 2017). Based on our comprehensive investigation, it has been determined that despite of having many folks uses, no scientific research has been conducted on the biological activities of the seed component through in vitro or in vivo experimentation. Hence, according to the phytochemical presence and folk medicinal uses, the present work aims to examine the possible anti‐diarrheal and anti‐pyretic activities of the crude methanolic extract of *C. maxima* seeds using a Swiss albino mice model.

## 2 Materials and Methods

### 2.1 Chemical

The source of loperamide was Square Pharmaceuticals Ltd., located in Gazipur, Bangladesh. The paracetamol sample was collected from Beximco Pharmaceuticals Ltd., located in Gazipur, Bangladesh. The methanol was obtained from the Sigma‐Aldrich (Humburg, Germany). Brewer's yeast, castor oil was procured from the local vendors. All of the chemicals and reagents utilized in this research were of analytical quality.

### 2.2 Plant Sample Collection and Identification

The *C. maxima* good quality seeds were obtained from a local plant nursery at the Bangladesh Forest Research Institute, located in Chattogram. The taxonomic authentication of the seeds was conducted by Dr. Sheikh Bokhtear Uddin, a taxonomist, Department of Botany, University of Chittagong, Chattogram, Bangladesh. The voucher specimen number is Accn No. SBU 5380.

### 2.3 Preparation and Quantitative Phytochemical Analysis of *C. maxima* Seed Extract

*C. maxima* seed were properly washed with water with care after collection. After that it was dried in shed for 1 week. Then it was processed as a fine powder using high‐capacity grinding machinery (origin: China, Model: 6P260, Voltage:220v, Power:5.0 hp) at the University of Chittagong in Department of Pharmacy. In two separate flasks, powdered seed were soaked in methanol solvent for 15 days, with regular shaking and stirring. A rotary evaporator was used to concentrate the mixture at a temperature of 60°C after it was filtered. The resultant crude extract (MCM) was stored in the fridge at 4°C temperatures until further analysis could be performed.

The preliminary phytochemical tests involve the testing of various chemical groups found in the extract. A limited amount of recently prepared MCM was utilized for an initial quantitative analysis to identify phytochemicals, including carbohydrates, alkaloids, tannins, glycosides, saponins, phytosterol, flavonoids, proteins, phenols, terpenes, fats, and fixed oils, employing established methodologies (Ahsan et al. 2023; Vishnoi 2009).

### 2.4 Test Animals and Treatment

The study utilized female Swiss Albino Mice with a weight range of 25–35 g. These mice were gathered from the animal resources center of ICDDR, B, Dhaka. In the animal facility of the Department of Pharmacy, University of Chittagong, they were habited in clean, dry cages with a 12‐h light/dark schedule and a temperature of 25°C ± 2°C. The animals were provided with a conventional laboratory diet and water “*ad libitum”*. Before start of the tests, the animals were allowed to acclimate for 3–4 days. Both before and during the trial, subjects went without food for 12 h. Animal handling and experiments were carried out following Helsinki Animal Ethics guidelines by The World Medical Association (WMA). Before initiating any treatment, the weight of each mouse was accurately measured, and the doses of the test samples and control materials were adjusted correspondingly. All drugs were prepared fresh prior to each experiment.

### 2.5 Anti‐Diarrheal Activity Inducing Castor Oil in Mice

The possible antidiarrheal activity of the methanolic crude extract of *Citrus maxima* seeds assessed by progressing diarrhea inducing castor oil in mice model described by Shoba (Shoba and Thomas 2001) and Franca (Franca et al. 2008) with modifications. Randomly 20 mice were chosen randomly and then distributed into 4 groups consisted of five mice each. Group I received a solution of 1%Tween 80 (10 mL/kg) via the oral route and marked as the control group. 1 mL of Tween‐80 was dissolved in 100 mL of normal saline (0.9% NaCl) to make 1% Tween‐80 solution (Erhirhie, Ekene, and Ajaghaku 2014). Group II was administered a conventional treatment of 50 mg/kg of loperamide via oral administration. Test groups consisting of Groups III and IV and were treated with doses of 200 and 400 mg/kg of MCM, respectively. All doses were freshly made before the procedure. Tween 80 in saline was used as a solvent for making all the doses including Loperamide. Before testing procedure, Mice were kept in a fasting phase for 18 h. They were administered the standard, control and test extract samples at 0 h. Then after 30 min, each mouse of every group was administered with 0.5 mL of castor oil through oral route to induce diarrhea. The animals were habited in separate places. The extent of induced diarrhea was examined for 4 h long. The total amount of feces (dry and wet droppings) was calculated with caution and it was compared with the negative control. The total number of wet droppings for the negative control was marked as 100%. The percentage inhibition of total defecation (solid and semi‐solid stool) and diarrhea were calculated using the following formulas: (Ezeja et al. 2012)

$$\text{Inhibition of defecation}\;\left(\%\right)=\frac{\text{Total number of feces in control}–\text{total number of feces in treated mice}}{\text{Total number of feces in control}}\times 100$$

$$\text{Inhibition of diarrhea}\;\left(\%\right)=\frac{\text{Total number of diarrheal feces in control}–\text{total number of diarrheal feces in treated mice}}{\text{Total number of diarrheal feces in control}\;}\times 100$$

### 2.6 Antipyretic Activity Inducing Pyrexia Using brewer's Yeast in Mice Model

The antipyretic potential was assessed through a previously described method (Zakaria et al. 2008). Twenty female Swiss albino mice were randomly selected and distributed into four groups, consisting of five mice in each group. They were kept fasted for 12 h and later administered as follows. Group‐I mice were given 1% Tween‐80 solution 10 mL/kg (control), Group‐II mice were treated with 150 mg/kg of dose of paracetamol (standard) while Groups‐III and IV mice were administered 200 and 400 mg/kg of methanolic extract through gastric gavage. Prior to testing, rectal temperatures of mice were measured carefully using a well lubricated bulb of a digital‐thermometer inserting in the rectum. Hyperpyrexia was induced in mice by administering subcutaneous injection of 20% brewer's yeast aqueous suspension of 10 mL/kg body weight of mice, in the back below the nape of the mice. After that, injected site was gently massaged in order to spread. At 20 h after the administration of yeast injection, the pre‐drug temperature was measured to determine the yeast's pyretic reaction. Animals with 0.5 degree or more rise in body temperature were used for this work. Each of the four groups of mice was administered control, standard and the investigated extracts 20 h after yeast injection. The temperature was recorded 1, 2, 3 and 4 h interval after the drug treatment.

### 2.7 Statistical Analysis

The study's findings were represented using the mean ± SEM (standard error of the mean) notation. The data were statistically analyzed using a one‐way analysis of variance (ANOVA) subsequently by a post hoc Dunnett's “*t*” test. The Statistical Package for Social Science (SPSS, version 16.0) was utilized for this purpose. Statistical significance was determined at the *p < 0.05, **p < 0.01 level, while very highly significant was determined at the ***p < 0.001 level.

## 3 Results

### 3.1 Preliminary Qualitative Phytochemical Analysis

This qualitative phytochemical investigation resulted positive about terpenoids, tannins, flavonoids, alkaloids, resin, glycosides, fat, and fixed oils inside the MCM sample. In addition, the negative result about phenols, saponin, phlobatanins, anthraquinones, steroid, cardiac glycosides, proteins and carbohydrates, was determined through examination (Ahsan et al. 2023).

### 3.2 Antidiarrheal Activity

Results regarding antidiarrheal activity of methanolic seeds extract of *C. maxima* (MCM) on castor oil induced diarrhea inhibition is shown in Table 1. It is obvious from the given data that MCM at both doses (200 and 400 mg/kg) showed a markedly significant (***p < 0.001) inhibition of diarrhea, in comparison to the control and standard group. At low dose (200 mg/kg) the effect was decreased moderately then the high dose (400 mg/kg). MCM therefore exhibited a dose dependent highly significant (***p < 0.001) antidiarrheal effect. The standard drug (loperamide 50 mg/kg) showed high anti‐diarrheal effect (***p < 0.001) than the MCM.

**TABLE 1:** Effect of MCM on castor‐oil induced diarrhea inhibition.

Treatment (mg/kg)	Mean no. of feces	Inhibition of defecation (%)	Mean no. of diarrheal feces	Inhibition of diarrhea (%)
Control‐1% Tween 80 solution (10 mL/kg)	20.80 ± 0.37	—	14.80 ± 0.49	—
Standard‐loperamide (50 mg/kg)	5.60 ± 0.24***	73.08	2.40 ± 0.24***	83.78
MCM (200 mg/kg)	9.80 ± 0.92***	52.88	4.40 ± 0.51***	70.27
MCM (400 mg/kg)	7.00 ± 0.55***	66.35	3.20 ± 0.37***	78.38

*Note:* All values are expressed as Mean ± SEM (*N* = 5).

Abbreviations: MCM = methanolic extract of *C. maxima* seeds, SEM = standard error of the mean.

*** *p* < 0.001 were regarded statistically significant compared to control.

### 3.3 Antipyretic Activity

Results obtained from antipyretic activity of methanolic seeds extract of *C. maxima* (MCM) on brewer's‐yeast induced hyperpyrexia inhibition is shown in Table 2. The standard drug (paracetamol 150 mg/kg) showed an excellent anti‐pyretic effect (**p < 0.01) at the 1st 2 h and it showed highly significant inhibition (***p < 0.001) at the last 2 h. Both doses (200 and 400 mg/kg) of MCM exhibited maximum significance as standard drug paracetamol at 3rd and 4th hours. At low dose (200 mg/kg) the effect was decreased in a moderate manner then the high dose (400 mg/kg). MCM thus showed a dose‐dependent significant antipyretic effect also.

**TABLE 2:** Effect of MCM on brewer's yeast induced pyrexia inhibition.

Treatment (mg/kg)	Pretreatment temperature (°F)		Posttreatment temperature (°F)			
	Normal	After yeast administration	1 h	2 h	3 h	4 h
Control‐1% Tween 80 solution (10 mL/kg)	97.52 ± 0.26	99.44 ± 0.29	100.84 ± 0.38	101.34 ± 0.49	101.92 ± 0.16	101.36 ± 0.38
Standard‐paracetamol (150 mg/kg)	96.84 ± 0.53	99.30 ± 0.28	98.24 ± 0.36**	97.44 ± 0.22**	96.86 ± 0.33***	96.24± 0.25***
MCM (200 mg/kg)	96.56 ± 0.18	97.80 ± 0.31	95.30 ± 1.01*	95.70 ± 0.76**	96.96 ± 0.29***	96.62 ± 0.54***
MCM (400 mg/kg)	95.90 ± 0.40	97.84 ± 0.39	93.26 ± 1.19**	93.46 ± 0.94**	95.14 ± 0.52***	96.20 ± 0.37***

*Note:* All values are expressed as Mean ± SEM (*N* = 5).

Abbreviations: MCM = methanolic extract of *C. maxima* seeds, SEM = standard error of the mean.

* *p* < 0.05.

** *p* < 0.01.

*** *p* < 0.001 were regarded statistically significant compared to control.

## 4 Discussion

Prior to the emergence of contemporary medical practices, individuals relied heavily on local plants and their components as a primary means of treating various illnesses. This technique has contributed to the establishment of a collective understanding regarding the diverse therapeutic characteristics exhibited by various plants and their components. Plants offer a wide array of diverse, intricate, and biologically significant chemicals that possess therapeutic properties. *Citrus maxima* , a member of the Rutaceae family, exhibits several ethno pharmacological applications (KunduSen et al. 2011). While previous research has explored several components of this particular plant, there has been a limited amount of investigation conducted on its seed. The present investigation lights on the crude methanolic seed extracts of *C. maxima* (MCM) in order to investigate its pharmacological efficacy as an antidiarrheal and antipyretic agent, hence suggesting its potential application in the field of medicine. Diarrheal disease frequently emerges as a prominent reason of illness and death, particularly in case of children residing in underdeveloped nations, hence presenting a significant healthcare challenge (Chitme, Chandra, and Kaushik 2004). The disease is characterized by a little or more discharge of semisolid or watery stool three or more times every day (Hirschhorn 1980; Snyder and Merson 1982). some synthetic drugs are existing for diarrheal treatment, though almost all of them have many side effects like irritable bowel movement etc. Anti‐motility and anti‐secretory agents, opioids and their derivatives are popularly used for treating diarrhea. Diphenoxylate, diphenoxin, and loperamide are commonly used opioid antidiarrheal drugs. Thus, continuous research for an alternative treatment with less side effect is still a conscious for the scientists. WHO has promoted research on the treatment and prevention against diarrheal illness through traditional ethno medical methods (Atta and Mouneir 2004). Hence, pharmacological floras' hold significant potential as a viable resource for the exploration and identification of novel anti‐diarrheal medicines. (Maikere‐Faniyo et al. 1989). The antidiarrheal activities of medicinal plants have been focused to the active presence of many bioactive compounds such as flavonoids, tannins, alkaloids, reducing sugars, saponins, sterols or terpenes. (Otshudi, Vercruysse, and Foriers 2000; Venkatesan et al. 2005). flavonoids have been recognized as having antidiarrheal activity due to their ability to reduce intestinal motility and hydroelectric discharges (Asmi et al. 2017; Rao et al. 1997) which were modified in this intestinal condition. Flavonoids have been demonstrated to suppress the intestinal secretion generated by prostaglandins E2, as investigated through in vitro and in vivo methods (de Medina et al. 1997). Therefore, the presence of flavonoids, alkaloids and terpenes in MCM might contribute their antidiarrheal activity. Previous research works suggested that castor oil was metabolized into the ricinoleic corrosive in the gut which was responsible for the looseness of the bowels production (Darracq et al. 2015). The administration of ricinoleate into the small digestive system enhanced the peristaltic action of the intestinal tract, resulting in a shift in the permeability of Na+ and Cl− ions in the intestinal mucosa. (Tariq et al. 2015). Discharge of endogenous prostaglandin was enhanced by ricinoleate (Beubler and Juan 1979; Sadraei et al. 2014). In the current study, loperamide, a widely used antidiarrheal drug was used as a reference standard which showed a maximum significant (***p < 0.001) decrease in defecation and diarrhea in the last 2 h in castor‐oil induced mice. Therefore, it could be suggested that, MCM at both doses (200 and 400 mg/kg) showed a maximum significant (***p < 0.001) dose dependent inhibition of diarrhea when compared with the control and standard treatment group.

A fever is characterized by an elevation in body temperature over the typical range of 36°C–37°C (99°F–100°F) in humans (Brazier, 2020). Typically, an increase in body temperature facilitates the individual's ability to overcome an infection. Nevertheless, there are instances when the temperature may escalate to an excessive level, so posing a significant risk and potentially resulting in consequences. (Brazier, 2020). An antipyretic is a pharmacological agent that is capable of diminishing fever. Antipyretics induce the hypothalamus to supersede an elevation in body temperature generated by prostaglandins. Subsequently, the physiological mechanisms of the human body engage in a process aimed at decreasing the elevated body temperature, hence leading to a decline in fever. The antipyretic agents most frequently utilized in clinical practice are ibuprofen and aspirin, both of which belong to the class of nonsteroidal anti‐inflammatory drugs (NSAIDs). While predominantly employed as analgesics for pain relief, both medications also possess antipyretic qualities. Additionally, paracetamol (acetaminophen), an analgesic with limited anti‐inflammatory effects, is commonly employed for its antipyretic properties (National Center for Biotechnology Information 2021). These NSAIDs inhibit COX‐2 production and thus inhibiting PGE_2_ biosynthesis to reduce the inflammation, as well as fever. However, they create toxic effects to the glomeruli, hepatic cells, heart muscle and cortex of the brain. Therefore, the investigation and development of novel agents possessing antipyretic and anti‐inflammatory potentials with improved safety‐efficacy profiles is a clinical need now (Pasin et al. 2010). Brewer's yeast is an external pyrogenous substance used in this research, that attach to the immunological protein known as the lipopolysaccharide binding protein (Ukwuani et al. 2012). The binding molecules interaction make the path for the generation and secretion of different endogenous cytokine factors, including Interleukin (IL‐1, IL‐6), and TNF‐α, which activate the arachidonic acid pathway, and eventually end up in the production and release of prostaglandin E_2_ (PGE_2_) (Gege‐Adebayo et al. 2013). Hyperpyrexia induced using brewer's yeast is recognized as pathogenic fever (Alzubier and Okechukwu 2011). The antipyretic response in animal model created by the crude extract (MCM) could be for the active presence of flavonoids, glycosides, tannins (Mutalik et al. 2003). The phytochemicals, tannins, and flavonoids were predominant in the inhibition of prostaglandin synthetase and cyclooxygenase or lipoxygenase, this mechanism helped in inhibition of induced pyrexia (Narayana et al. 2001). Terpenoids also have antipyretic activity. The presence of these phytochemicals in this investigated extract might contribute to their antipyretic activity. In the present study paracetamol started ameliorating the pyrexia from 1 h after its administration which significantly (***p < 0.001) reduced the pyrexia after 4 h of its administration. Both doses (200 and 400 mg/kg) of MCM exhibited maximum significance as standard drug paracetamol at 3rd and 4th hours. So, it is possible that blocking the synthesis of PGs like paracetamol is how antipyretic activity works (Chandrasekharan et al. 2002) and inhibition of any of the several multi‐processes or mediators emphasizing the pathogenesis of fever, might bring about antipyretic (Srivastava et al. 2013).

## 5 Conclusion

The plant under investigation, *C. maxima* , is a commonly utilized ethno‐pharmacological medicinal plant. The methanolic seed extract of this plant shows considerable potential as an agent for combating diarrhea and reducing fever. Nevertheless, it is possible to expand upon this research in order to understand the underlying mechanism of action of the examined extracts. This would involve identifying and isolating the specific functional chemicals that may be responsible for the reported pharmacological effects. Nevertheless, further research is warranted to elucidate the underlying mechanism of action of the examined extracts. This would involve identifying and isolating the specific bioactive compounds responsible for the observed pharmacological effects. Such inquiry has the potential to lead to the discovery of new therapeutic molecules with enhanced pharmacological properties and improved safety profiles.

## Author Contributions

**Nazratun Noor Maria:** conceptualization (lead), data curation (lead), formal analysis (lead), funding acquisition (equal), investigation (lead), methodology (lead), project administration (lead), resources (lead), software (lead), validation (equal), visualization (lead), writing – original draft (lead), writing – review and editing (equal). **Ayesha Akter Jasmin:** formal analysis (supporting), investigation (supporting), project administration (supporting). **Saurav Singha:** formal analysis (supporting), investigation (supporting), project administration (supporting). **Umme Tahmida:** formal analysis (supporting), investigation (supporting), project administration (supporting). **Tanveer Ahsan:** formal analysis (supporting), funding acquisition (equal), supervision (lead), writing – review and editing (equal).

## Conflicts of Interest

The authors declare no conflicts of interest.

## Data Availability

The data that support the findings of this study are available on request from the corresponding author. All data and materials are contained and described within the manuscript. The plant's materials for the study were identified and voucher specimens are deposited at Pharmacy Department of University of Chittagong Accn No. SBU 5380.
