# Correction to “Investigation on Anti‐Diarrheal and Antipyretic Activities of *citrus maxima* Seeds in Swiss Albino Mice Model”

**DOI:** 10.1002/fsn3.70210

**Published:** 2025-04-25

Ashan, T. , Maria, N.N. , Jasmin, A.A. , Tahmida, U. , and Singha, S. (2025), Investigation on Anti‐Diarrheal and Antipyretic Activities of citrus maxima Seeds in Swiss Albino Mice Model. Food Sci Nutr, 13: e4631. 10.1002/fsn3.4631 39803243 PMC11717002

In the originally published article, the order of authors was incorrect. The correct order is: Nazratun Noor Maria, Ayesha Akter Jasmin, Umme Tahmida, Saurav Singha, and Tanveer Ahsan

The citation should read as Maria, N.N., Jasmin, A.A., Tahmida, U., Singha, S., and Ahsan, T. (2025), Investigation on Anti‐Diarrheal and Antipyretic Activities of citrus maxima Seeds in Swiss Albino Mice Model. Food Sci Nutr, 13: e4631. https://doi.org/10.1002/fsn3.4631

The online version has been corrected accordingly.

We apologize for this error.

