# Correction to “Investigation on Anti‐Diarrheal and Antipyretic Activities of *Citrus maxima* Seeds in Swiss Albino Mice Model”

**DOI:** 10.1002/fsn3.70569

**Published:** 2025-07-25

Maria, N. N., A. A. Jasmin, U. Tahmida, S. Singha, and T. Ahsan. 2025. Investigation on Anti‐Diarrheal and Antipyretic Activities of *Citrus maxima* Seeds in Swiss Albino Mice Model. *Food Science & Nutrition* 13: e4631. https://doi.org/10.1002/fsn3.4631.

In paragraph 4 of the “Materials and Methods” section, the text “Animal handling and experiments were carried out following Helsinki Animal Ethics guidelines by The World Medical Association (WMA)” was missing the ethical approval information. This should have read: “Animal handling and experiments were carried out following Helsinki Animal Ethics guidelines by The World Medical Association (WMA) and the approving institution is Department of Pharmacy, University of Chittagong and the approval reference number: ERB‐FBSCU‐20230718‐(1).”

We apologize for this unintended omission.

