# Correction to Investigation on Anti‐Diarrheal and Antipyretic Activities of *Citrus maxima* Seeds in Swiss Albino Mice Model

**DOI:** 10.1002/fsn3.70746

**Published:** 2025-07-28

Maria NN , Jasmin AA , Tahmida U , Singha S , Ahsan T . 2025. “Investigation on Anti‐Diarrheal and Antipyretic Activities of citrus maxima Seeds in Swiss Albino Mice Model.” Food Science & Nutrition 13, no. 4: e4631.39803243 10.1002/fsn3.4631PMC11717002

The published article mistakenly did not include all details of the ethical approval for animal experiments included in the article. The name of the approving institution is Department of Pharmacy, University of Chittagong, and the approval reference number is ERB‐FBSCU‐20230718‐(1).

In paragraph 4 of the “Materials and Methods” section, the text “Animal handling and experiments were carried out following Helsinki Animal Ethics guidelines by The World Medical Association (WMA).” was missing the ethical approval information. This should have read: “Animal handling and experiments were carried out following Helsinki Animal Ethics guidelines by The World Medical Association (WMA) and the approving institution is Department of Pharmacy, University of Chittagong and the approval reference number: ERB‐FBSCU‐20230718‐(1).”

We apologize for this unintended omission.

